# Supplementary material for: Health Education Intervention on Hearing Health Risk Behaviors in College Students
Source: Int J Environ Res Public Health. 2021 Feb 6;18(4):1560. doi: 10.3390/ijerph18041560 (PMC7914527; doi:10.3390/ijerph18041560)
Supplement: Supplementary file 1 [file ijerph-18-01560-s001.pdf]

Table S1 Comparison of hearing health knowledge and belief between different groups at various intervention stages in male students (n=241).

Variable	Groups	Before intervention T1 (Mean±SD)	After intervention T2 (Mean±SD)	T3 (Mean±SD)	*p* Time group	Control and Intervention	Interaction
Score of hearing health knowledge	Control	30.37±6.24	31.40±8.10	31.57±9.94	0.108	0.896	1.369
	Intervention	29.33±6.77	32.80±7.47	31.28±11.65			
	t	0.611	-1.325	0.353			
	p	0.547	0.297	0.832			
Score of hearing health belief	Control	164.57±18.14	163.02±19.07	163.69±21.45	0.552	0.990	0.314
	Intervention	163.87±16.13	166.34±18.32	161.59±23.74			
	t	0.034	-2.803	2.68			
	p	0.99	0.353	0.447			
Perceived susceptibility	Control	13.05±3.50	12.92±3.63	13.61±3.47	0.305	0.288	0.529
	Intervention	13.77±3.50	13.28±3.48	13.92±3.69			
	t	-0.863	-0.316	-0.186			
	p	0.123	0.582	0.741			
Perceived severity	Control	37.84±4.86	36.96±6.66	36.79±8.21	0.790	0.738	0.392
	Intervention	37.67±4.68	38.10±5.55	36.20±9.37			
	t	0.062	-1.269	0.468			
	p	0.936	0.215	0.731			
Perceived benefits	Control	30.81±4.53	30.59±4.90	29.43±6.13	0.129	0.184	0.778
	Intervention	30.11±3.96	30.61±4.16	28.44±7.31			
	t	0.760	0.450	1.169			
	p	0.280	0.549	0.256			
Perceived	Control	24.92±6.83	24.19±7.37	23.18±7.70	0.105	0.504	0.749

barriers	Intervention	23.93±6.62	24.33±7.16	22.26±8.17			
	t	1.105	0.058	0.541			
	p	0.307	0.960	0.665			
Cues to action	Control	28.69±6.03	28.93±6.17	29.88±6.29	**0.005**	0.808	0.528
	Intervention	28.56±4.85	29.59±4.55	29.95±6.72			
	t	-0.354	-0.711	0.580			
	p	0.699	0.443	0.568			
Perceived self-efficacy	Control	29.26±5.34	29.42±5.63	30.80±5.98	0.354	0.441	0.619
	Intervention	29.82±5.08	30.44±5.78	30.82±6.79			
	t	-0.676	-1.016	0.108			
	p	0.423	0.262	0.913			

Note: All values are Means±SD(Standard Deviation); T1, before the intervention; T2, at the intervention cessation phase; T3, 3 months after the intervention cessation (maintenance phase);

Significant differences between groups were determined by using Repeated Measures ANOVA and t-test with age and grades being adjusted.

Boldface indicates statistical significance ($p<0.05$)

Table S2 Comparison of hearing health knowledge and belief between different groups at various intervention stages in female students (n=589).

Variable	Groups	Before intervention T1 (Mean±SD)	After intervention T2 (Mean±SD)	T3 (Mean±SD)	*p* Time group	Control and Intervention	Interaction
Score of hearing health knowledge	Control	31.51±5.17	32.96±6.48	33.46±7.36	**0.028**	**0.018**	**0.011**
	Intervention	31.01±5.47	34.24±4.23	34.98±6.16			
	t	0.251	-1.23	-1.449			
	p	0.586	**0.007**	**0.012**			
Score of hearing health belief	Control	171.08±13.98	168.41±16.30	168.67±14.97	0.231	0.535	0.109
	Intervention	168.08±14.67	167.98±15.03	169.40±16.95			
	t	2.265	0.393	-0.722			
	p	0.067	0.77	0.606			
Perceived susceptibility	Control	13.77±3.30	13.13±4.00	14.03±3.44	0.198	0.100	0.870
	Intervention	13.41±2.98	12.87±3.33	13.55±3.55			
	t	0.36	0.303	0.47			
	p	0.18	0.331	0.122			
Perceived severity	Control	38.86±4.42	38.64±5.28	38.75±5.01	0.596	0.475	0.067
	Intervention	38.11±4.74	39.25±5.23	39.13±5.39			
	t	0.511	-0.671	-0.484			
	p	0.197	0.14	0.286			
Perceived benefits	Control	31.06±4.05	31.13±4.32	30.56±4.39	0.752	0.479	0.843

	Intervention	30.65±4.15	30.94±4.56	30.44±4.98			
	t	0.345	0.162	0.075			
	p	0.333	0.675	0.856			
Perceived barriers	Control	25.84±5.97	24.98±6.88	22.64±6.73	0.538	0.116	0.071
	Intervention	24.73±5.79	23.93±6.37	23.26±7.06			
	t	1.015	1.143	-0.221			
	p	**0.045**	**0.045**	0.71			
Cues to action	Control	30.11±4.54	30.26±5.57	30.75±4.54	0.492	0.429	0.452
	Intervention	30.48±4.30	30.01±5.32	30.84±5.04			
	t	-0.522	0.114	-0.306			
	p	0.168	0.807	0.464			
Perceived self-efficacy	Control	31.43±4.63	30.27±4.94	31.94±4.60	0.939	0.703	**0.023**
	Intervention	30.70±4.58	30.98±4.63	32.18±4.84			
	t	0.557	-0.658	-0.256			
	p	0.161	0.11	0.533			

Note: All values are Means±SD (Standard Deviation); T1, before the intervention; T2, at the intervention cessation phase; T3, 3 months after the intervention cessation (maintenance phase);

Significant differences between groups were determined by using Repeated Measures ANOVA and t-test with age and grades being adjusted.

Boldface indicates statistical significance ($p<0.05$).

Table S3 Baseline characteristics of hearing health behaviors between different groups in male and female students (n=830).

Variables	Male		*p*	Female		*p*
	Control (n=180)	Intervention (n=61)		Control (n=231)	Intervention (n=358)	
**Average frequency of using headphones**			0.343			0.782
<3 times/day	124（68.9）	38（62.3）		166（71.9）	261（72.9）	
≥3 times/day	56（31.1）	23（37.7）		65（28.1）	97（27.1）	
**Average duration of using headphones**			0.645			0.875
<30 minutes/time	62（34.4）	23（37.7）		103（44.6）	162（45.3）	
≥30 minutes/time	118（65.6）	38（62.3）		128（55.4）	196（54.7）	
**Probability of increasing headphone volume in noisy environment**			0.813			0.716
＜50%	62（34.4）	20（32.8）		99（42.9）	148（41.3）	
≥50%	118（65.6）	41（67.2）		132（57.1）	210（58.7）	
**Volume of the headphones of the total volume**			0.605			**0.014**
<40%	133（73.9）	43（70.5）		187（81.0）	258（72.1）	
≥40%	47（26.1）	18（29.5）		44（19.0）	100（27.9）	
**Using in-ear type headphones**			0.586			0.967
No	102（56.7）	37（60.7）		159（68.8）	247（69.0）	

Yes	78（43.3）	24（39.3）		72（31.2）	111（31.0）	
**Sleeping with headphones for listening to music or radio**			0.210			0.125
No	108（60.0）	31（50.8）		133（57.6）	183（51.1）	
Yes	72（40.0）	30（49.2）		98（42.4）	175（48.9）	

Note: All values are n (%), and chi-square test was used to analyze the differences between intervention group and control group; Boldface indicates statistical significance ($p<0.05$).
